# Autism Spectrum Disorder: High-Resolution Elucidation of Mitochondrial Dysregulation in Larval Zebrafish Gut

**DOI:** 10.3390/cells15141305

**Published:** 2026-07-21

**Authors:** Johanna A. Coetzee, Lesha Pretorius, Janica Theron, Angela Latakgomo, Carine Smith

**Affiliations:** 1 Experimental Medicine, Department of Medicine, Faculty of Medicine and Health Sciences, Stellenbosch University, Cape Town 7500, South Africa; 2 Tygerberg Electron Microscopy Unit, Central Analytical Facilities, Stellenbosch University, Cape Town 7500, South Africa

**Keywords:** redox imbalance, mitochondrial respiration, mitophagy-lysosomal axis, autophagy, fission, PINK1, LC3B, transmission electron microscopy, confocal microscopy

## Abstract

**Highlights:**

**What are the main findings?**

- Mitochondrial dysfunction in autism spectrum disorder (ASD) is multifactorial and not only linked to electron transport chain pathology.
- Mitochondrial morphometric abnormalities are illustrated in the ASD-like gut for the first time.

**What is the implication of the main findings?**

- Consideration of the gut as part of a multicompartmental management approach is warranted in ASD.

**Abstract:**

Although gastrointestinal distress is both common and debilitating in individuals with autism spectrum disorder (ASD), underpinning mechanisms—and therefore effective management strategies—are not fully elucidated. The current study employed well-established valproic acid ASD model in larval zebrafish to more comprehensively characterize mitochondrial dysregulation. Whole body redox status and mitochondrial respiration, as well as protein expression in the mitophagy-lysosomal axis in the mid-intestine was assessed. In addition, high-resolution microscopy of the gut was used to assess mitochondrial morphometrics and distribution. Redox imbalance was evident from increased oxygen radical levels and decreased endogenous antioxidant capacity, as well as depression of whole body mitochondrial respiration. Upregulation of endo-lysosomal pathway markers (Rab5, LAMP1) together with reduced expression in mitophagy and autophagy markers (PINK1, LC3B) suggests a potential impairment of canonical mitophagy in the ASD-like gut. High-resolution imaging further revealed smaller, more circular mitochondria, indicative of a morphological fission bias. Abnormal mitochondrial distribution patterns were also evident. Together, current data points to primary mitochondrial dysfunction as a potential feature of the ASD-like gut. Furthermore, data suggest potential insufficiencies in mitochondrial recycling as potential role player in ASD-associated (gut) mitochondrial pathology.

## 1. Introduction

As the prevalence of autism spectrum disorder (ASD) increases [1], the undermanagement of non-neurological ASD-associated symptomologies is gaining attention [2,3,4,5,6]. Addressing this gap by increasing our understanding of ASD-associated cellular pathophysiology is thus a priority for therapeutic target identification and symptomatic management. In this context, the use of preclinical ASD models is ideal for understanding mechanistic pathologies and systemic dysregulation. The valproic acid (VPA) model mimics idiopathic ASD to a large extent [7] and is based on reports from the clinical setting which links maternal gestational use of VPA for the management of epilepsy, to an increased risk of ASD diagnosis in in utero-exposed offspring [8]. In preclinical models, VPA exposure during early development induces an ASD-like phenotype in various species (zebrafish, rodents, monkeys), providing a reliable translational research tool [7]. In addition to the consistent recapitulation of behavioural features, this model also demonstrates persistence of molecular abnormalities observed in early developmental stages into adulthood, such as mitochondrial dysfunction, which has been repeatedly (but superficially) reported [9,10,11,12,13,14].

Poorer gut muscle tone and peristaltic contractility have been reported in the VPA model in both larval zebrafish [15] and rodents [16]. In the zebrafish study, which emanated from our group, we further illustrated a significant increase in lysosomal abundance in the gut, which lead to the hypothesis that ASD-associated mitochondrial dysfunction may—at least in part—contribute to the observed gut symptomologies. Indeed, the association between gut symptoms and ASD through mitochondrial dysfunction is particularly compelling, given the high prevalence of gut-associated comorbidities (9–91%) in ASD [3,17,18], the well-established association between mitochondrial dysfunction and gut pathology [19,20,21,22,23], and the known presence of mitochondrial dysfunction in ASD.

Mitochondrial dysfunction (and thus increased oxidative stress) in the gut has wide-ranging downstream consequences, including potentially impaired epithelial barrier integrity, altered smooth muscle tone and microbial dysbiosis [22]. Apart from the significantly increased prevalence of classical mitochondrial disease reported in ASD (~5% vs. ~0.01% in the general population), 30–80% of individuals with ASD exhibit biomarkers of mitochondrial dysfunction [24]. Mostly, studies investigating aspects of the electron transport chain (ETC) suggest altered activity and/or expression of one or more ETC complexes across multiple tissues, including postmortem brain tissue [25,26], skeletal muscle biopsies [27], buccal epithelium [28,29], peripheral blood cells [30], immune cell-like granulocytes [31], lymphocytes [32] and cecum biopsies [23]. Beyond abnormalities in the ETC, alterations in several biomarkers commonly associated with mitochondrial dysfunction—including lactate, pyruvate, alanine, creatine kinase, carnitine—have also been reported in clinical samples in the ASD context [24,33]. In terms of mitochondrial dynamics specifically, increased expression of the fission proteins (Fis1 and Drp1) and decreased expression of the fusion proteins (Mfn1, Mfn2, and Opa1) have been reported in postmortem ASD brain tissue [25]. However, published studies focused on mitochondrial dysfunction—particularly mitochondrial dynamics and mitophagy—in the ASD gut are limited.

Therefore, the current study employed the VPA model in larval zebrafish to further probe the links between redox imbalance and mitochondrial dysregulation in the gut. Specifically, the relative contribution of the PINK1/Parkin-mitochondrial-lysosomal axis as mechanistic role player in ASD-associated mitochondrial dysfunction was investigated. In addition, high-resolution morphometric analysis of epithelial mitochondria was performed for the first time in the ASD gut context.

## 2. Materials and Methods

### 2.1. Ethical Considerations

All experimental protocols were approved by the Stellenbosch University Research Ethics Committee for Animal Care and Use (ACU-2025-27020). Wild-type zebrafish (*Danio rerio*) embryos were obtained from the Zebrafish Research Unit (Experimental Medicine, Department of Medicine, Stellenbosch University, Cape Town, South Africa) and maintained in embryo medium (E3; 5 mM NaCl, 0.17 mM KCl, 0.33 mM CaCl_2_•2 H_2_O, 0.33 mM MgSO_4_•7 H_2_O, 1.3 × 10^−5^ % *^w^*/*_v_* methylene blue in reverse osmosis water) at ambient conditions of 28 °C and 40–60% humidity, with a 14:10 h light: dark cycle.

### 2.2. Induction of an ASD-like Phenotype

ASD-like phenotype induction was achieved as previously described [15,34] (also refer to Appendix A, Table A1). Briefly, embryos were randomly assigned to experimental groups (control and ASD-like). The embryos assigned to the ASD-like group were immersed in 37.5 µM VPA (Sigma-Aldrich, Darmstadt, Germany, P4543) from 4 to 52 h post fertilization (hpf) and subsequently maintained in E3 until 7 days post fertilization (dpf) (experimental endpoint). At experimental endpoint, larvae were euthanized by tricaine overdose (400 µg/mL) and processed according to the specifications of the respective postmortem analyses. All employed protocols employed were optimized to minimize distress and no unexpected adverse events occurred (monitored according to OECD guidelines).

### 2.3. Whole Body Redox Assays

All biological replications originated from independent spawning events. All biochemical assays were performed in triplicate.

#### 2.3.1. Trolox Equivalent Antioxidant Capacity (TEAC) Assay

The TEAC assay was performed in pooled samples (50 larvae per sample, *n* = 15–16 per group) as previously described [35]. Absorbances were related to total protein content and data reported as a percentage of control.

#### 2.3.2. Superoxide Dismutase (SOD) Activity

SOD activity was assessed in pooled larvae (50 larvae per sample, *n* = 15–16 per group). Samples were snap-frozen in Tris-HCl buffer (pH 7.4) and mechanically homogenized using a Bead Ruptor Elite (OMNI International, Kennesaw, GA, USA). Homogenates were centrifuged at 14,000× *g* for 5 min at 4 °C, and the supernatants collected for analysis. Total SOD activity was quantified using a commercially available assay kit (Sigma-Aldrich, Darmstadt, Germany, CS0009) according to the manufacturer’s instructions. Absorbances (read at 450 nm) were related to total protein content and data reported as a percentage of control.

#### 2.3.3. Reactive Oxygen Species (ROS) Assay

For ROS analysis, larvae (*n* = 10 per group) were rinsed with ice cold PBS and homogenized in cold homogenisation buffer (20 mM HEPES, 320 mM Sucrose, 0.1 mM MgCl_2_, 0.5 mM PMSF at pH 7.4). The homogenate was centrifuged at 12,000× *g* for 30 min at 4 °C, supernatant collected and 20 µL transferred to 96-well plate. This was followed by the addition of 100 µL PBS (pH 7.4) and 8.3 µL 2′-7′-dichlorofluorescin diacetate (DCFH-DA; Sigma-Aldrich, Darmstadt, Germany, 35845) solution dissolved in DMSO (0.1 mg/mL). The plate was incubated at 37 °C for 30 min protected from light. Fluorescence (read at 535 nm) were related to total protein content and data reported as a percentage of control.

#### 2.3.4. Mitochondrial Superoxide

Larvae (*n* = 8 per group) were incubated in 2.5 µM MitoSOX™ Green (Thermo Fisher Scientific, Waltham, MA, USA, M36006) solution for 30 min at 28.5 °C. After staining, larvae were washed (3× with E3). Anesthetized individual larvae were oriented laterally for optimal visualization of the liver (ROI). Imaging was performed using a Nikon^®^ ECLIPSE Ti2 (Tokyo, Japan) inverted fluorescence microscope. Images were acquired using NIS-Elements D software (v5.30.02) and subsequently analyzed in ImageJ software (v1.54p).

### 2.4. Mitochondrial Respiration

A high-resolution O2k-FluoRespirometer (Oxygraph O2k; Oroboros Instruments GmbH, Innsbruck, Austria) was used to measure mitochondrial respiration via specialized substrate–uncoupler–inhibitor–titration (SUIT) protocol (SUIT_001_O2_pfi_D002) (Table A2 in Appendix A) in pooled larvae (100 larvae per sample, *n* = 4 per group). Euthanized larvae were washed with PBS and resuspended in 50 µg/mL digitonin solution. Samples were placed on ice for 20 min and frequently agitated to ensure adequate tissue permeabilization. Permeabilized larvae were washed and resuspended in 2 mL mitochondrial respiration media (MiRO5) and transferred to the oxygraphy chambers. Prior to the experiments, the chambers were set to 28 °C and hyperoxygenated (to approximately 500 µmol/L O_2_). Oxygen consumption was recorded throughout the protocol using DatLab software (v7.4.0.4). Data were expressed as pmol/s/L, normalized to estimated pooled larval weight (0.04 mg per larvae) and corrected for residual oxygen consumption (Rox).

### 2.5. Gut-Specific Parameters

All images were quantified with the investigator blinded to group allocation.

#### 2.5.1. Lysosomal Staining

Neutral red staining was performed as previously described [36]. Briefly, larvae (*n* = 30–33 per group) were immersed in 2 µg/mL neutral red solution for 5 h. Anesthetized zebrafish larvae were imaged at 100× magnification using Nikon^®^ ECLIPSE Ti2 inverted microscope (Tokyo, Japan) with image acquisition in NIS-Elements D v 5.30.02 software. Images were quantified with ImageJ software (version 1.54p).

#### 2.5.2. Immunofluorescent Staining

Fixed zebrafish larvae (*n* = 12–17 per group) were embedded in 2% low-melting-point agarose and sectioned (70 µm) in the transverse plane using a VT 1000 S vibratome (Leica Microsystems, Vienna, Austria) (speed, 0.70 mm/s; frequency, 6 Hz; amplitude, 1 mm). Whole larvae and sections were subjected to immunofluorescent labelling as previously described [35]. Sequential primary and secondary antibody incubations utilizing the following antibodies: 1:200 LC3B (Invitrogen, Carlsbad, CA, USA, PA1-46286), 1:50 TOMM20 (Invitrogen, Carlsbad, CA, USA, HPA011562), 1:20 PINK1 (Invitrogen, Carlsbad, CA, USA, MA5-11153), 1:20 Parkin (Invitrogen, Carlsbad, CA, USA, 390900), 1:50 LAMP1 (Invitrogen, Carlsbad, CA, USA, 14-1071-82), 1:20 Rab5 (BD bioscience, San Jose, CA, USA, 610724), 1:250 Alexa Fluor 488 donkey anti-mouse (Invitrogen, Carlsbad, CA, USA, A21202), 1:250 Alexa Fluor 594 donkey anti-rabbit (Life technologies; Carlsbad, CA, USA, A21207), 1:250 Alexa Fluor 405 donkey anti-rat (Invitrogen, Carlsbad, CA, USA, A48268) and 1:250 Alexa Fluor 488 anti-rabbit (Invitrogen, Carlsbad, CA, USA, A21202).

For identification of the mid-intestinal region of the gut, brightfield images were overlaid with immunostained images to allow for standardized demarcation of the mid-intestinal region relative to landmarks (swim bladder and cloaca). Regions of interest were standardized for size to ensure that area was not a confounder in quantification of fluorescence. Fluorescence data was expressed as integrated density (mean grey value/area).

#### 2.5.3. Whole-Mount Fluorescent Imaging

Laterally orientated larvae were imaged at 100× magnification using Nikon^®^ ECLIPSE Ti2 inverted microscope (Tokyo, Japan) with image acquisition in NIS-Elements D v 5.30.02 software. Images were quantified with Image J (v1.54p).

#### 2.5.4. Confocal Microscopy

Immunolabelled sections were imaged using a Zeiss LSM 780 microscope (CAF Microscopy Unit, RRID:SCR_023596) at 200× and 630× magnification. ZEISS ZEN 2012 (black edition, v8.1) software was used for image acquisition and further image processing (maximum intensity projection).

#### 2.5.5. Electron Microscopy

Larvae (*n* = 6–7 per group) were fixed (2.5% glutaraldehyde and 4% formaldehyde in 0.1 M phosphate buffer, pH 7.4) for 24 h at 4 °C. Samples were washed in 0.1 M phosphate buffer, and post-fixed using an adapted Osmium–Thiocarbohydrazide–Osmium (OTO) protocol [37]. In short, sequential incubation in 4% osmium tetroxide/3% potassium ferricyanide (1:1) for 60 min at 4 °C, 20 min in thiocarbohydrazide (room temperature) and 30 min in 2% aqueous osmium tetroxide (room temperature) were performed. Samples were then incubated overnight in 1% uranyl acetate at 4 °C. All preceding steps were separated by three 5 min wash steps in distilled water. Samples were dehydrated through a 5-step graded ethanol series (20–100%, 10 min each at 4 °C), followed by 100% ethanol and subsequent acetone incubation (10 min each, room temperature). Resin infiltration was performed using increasing concentrations of EPON in acetone (50% for 2 h; 75% for 2 h; 100% overnight). Samples were embedded in fresh 100% EPON and polymerised at 60 °C for 48 h. Samples were sectioned with Leica UC7 ultramicrotome (Leica Microsystems, Vienna, Austria) fitted with a 45° diamond knife. Gut samples were trimmed to isolate the mid-intestine, identified by the presence of goblet cells [38,39], and sectioned at 110 nm. Sections were collected on silicon wafers and imaged using an Apreo VolumeScope SEM (Thermo Fisher Scientific, Eindhoven, The Netherlands) with Xt Microscopy software (Thermo Fisher Scientific, Eindhoven, The Netherlands).

Mitochondrial morphometrics included mitochondrial number (individual mitochondria within 10 cells were quantified and the average calculated), mitochondrial area (*n* = 60 mitochondria), and mitochondrial aspect ratio (*n* = 60 mitochondria).

### 2.6. Statistical Analysis

Visualization and statistical analyses of all data were completed utilizing GraphPad Prism version 9.4.0 (http://www.graphpad.com, San Diego, CA, USA). The ROUT outlier test (Q = 1%) was employed to remove outliers. The Shapiro–Wilk test was used to assess the normality of data distribution. For normally distributed data, Student’s *t*-tests (equal group variances) or Welch’s *t*-tests (unequal group variances) were performed. Alternatively, if data exhibited a non-parametric distribution, a Mann–Witney test was performed. Respirometry data was analyzed with a two-way ANOVA and Šídák’s post hoc testing to correct for multiple comparisons. A *p*-value < 0.05 was considered statistically significant. Quantitative data were presented as individual scatter plots with mean ± standard deviation (SD) for normally distributed data or median ± 95% confidence intervals (CI) for non-parametric data.

## 3. Results

### 3.1. Whole Body Indicators Demonstrate Increased Oxidative Stress and Mitochondrial Dysfunction

**Figure 1:**
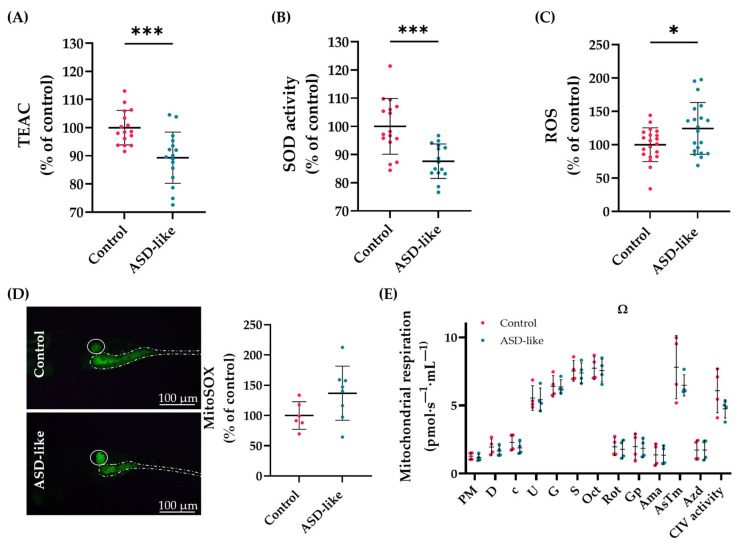
Characterization of redox imbalance and associated mitochondrial respiratory deficits in an ASD-like zebrafish larval model. Whole body redox profiling including (**A**) total antioxidant capacity, (**B**) superoxide dismutase activity and (**C**) reactive oxygen species assessments. Mitochondrial-specific parameters included assessment of (**D**) mitochondrial superoxide (MitoSOX) with liver ROI indicated by white circle and the gut by the white dotted line (Images captured at 40× magnification scale bar represents 100 µm) (**E**) whole body mitochondrial respiration. All data are presented as mean ± SD. Samples consisted of (**A**–**C**) 50 pooled larvae with *n* = 15–16, (**D**) *n* = 5–8 and (**E**) 100 pooled larvae with *n* = 4. Statistical analysis: (**A**–**D**) Welch’s and (**E**) two-way ANOVA with Šídák’s multiple comparisons test; * *p* < 0.05, *** *p* < 0.001 and Ω indicates a main ANOVA effect for VPA-exposure (Ω, *p* < 0.05). Abbreviations: Ama, antimycin A; AsTm, ascorbate and tetramethyl phenylenediamine (TMPD); c, cytochrome c; D, adenosine diphosphate (ADP); G, glutamate; Gp, glycerophosphate; Oct, octanoylcarnitine; PM, pyruvate and malate; Rot, rotenone; S, succinate; SOD, super oxide dismutase; TEAC, Trolox equivalent antioxidant capacity; Azd, sodium azide; U, uncoupler (carbonyl cyanide m-chlorophenylhydrazone).

Whole body analysis illustrated deficits in both total antioxidant capacity (Figure 1A) and superoxide dismutase activity (Figure 1B), as well as increases in ROS (Figure 1C) in the ASD-like phenotype. Similarly, mitochondrial-specific ROS (quantified at 40× magnification) showed a trend toward upregulation in the ASD-like liver (Figure 1D, *p* = 0.069). In terms of mitochondrial respiration, high-resolution respirometry revealed an overall decrease in mitochondrial oxygen consumption across the electron transport chain (main ANOVA effect of ASD-like phenotype; *p* < 0.05; Figure 1E).

### 3.2. ASD-Associated Mitochondrial and Lysosomal Dysfunction Observed in the Gut

**Figure 2:**
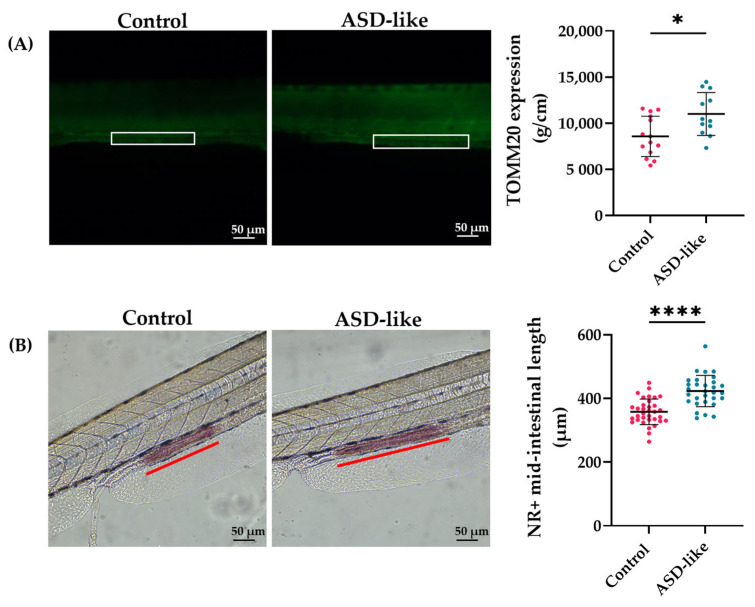
Mitochondrial and lysosomal abundance in an ASD-like zebrafish larval gut. Representative micrographs of (**A**) TOMM20 expression in the gut (green, *n* = 12–14) and (**B**) neutral red positive stained mid-intestinal region (red, *n* = 30–33) with corresponding quantitative data presented as mean ± SD. Scale bar represents (**A**) 50 µm captured at 100× magnification and (**B**) 100 µm captured at 100× magnification. The white rectangles demarcate the gastrointestinal region quantified in immunofluorescent images and red lines span the neutral red positive stained area. Statistical analysis: Welch’s *t*-test; * *p* < 0.05; **** *p* < 0.0001.

To assess mitochondrial localisation and abundance within the gut tissue compartment, immunofluorescent staining targeting TOMM20—an outer mitochondrial membrane marker—was performed. A significant increase in TOMM20 expression was observed in the ASD-like phenotype (Figure 2A), while an increase in the length of neutral red-positive stained in the gut region (Figure 2B) suggested an expansion of lysosome-rich compartments in the gut.

**Figure 3:**
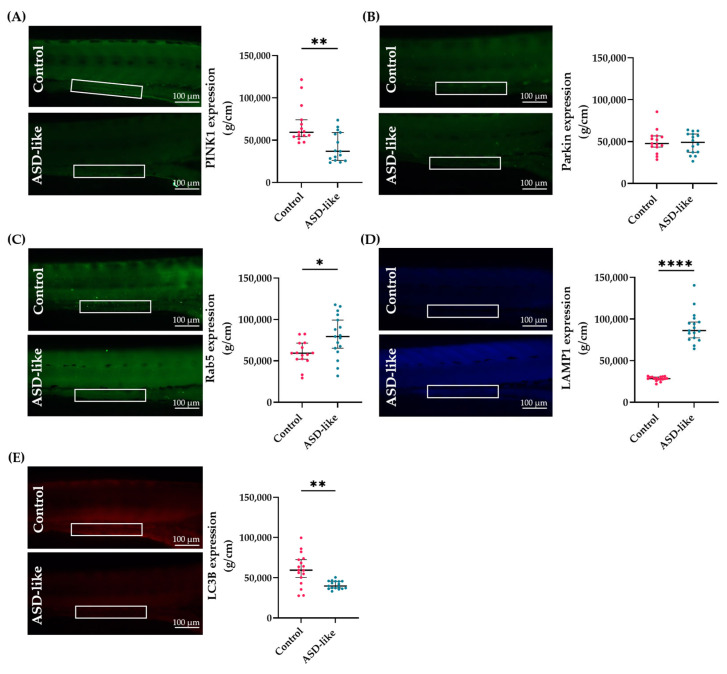
Expression of key proteins in the PINK1/Parkin-mitophagy-lysosomal axis in an ASD-like zebrafish larval gut. Representative micrographs and qualitative data of (**A**) PINK1, (**B**) Parkin, (**C**) Rab5, (**D**) LAMP1 and (**E**) LC3B protein expression. Statistical analysis: Mann–Whitney median ± 95% CI, *n* = 12–17; * *p* < 0.05; ** *p* < 0.01; **** *p* < 0.0001. Scale bar = 100 µm. Abbreviations: LAMP1, Lysosome-associated membrane protein 1; LC3B, Microtubule-associated protein 1A/1B-light chain 3-phosphatidylethanolamine conjugate; PINK1, PTEN-induced kinase 1; Rab5, Ras-associated protein 5.

To elucidate mechanisms at play, relative abundance of role players in the PINK1/Parkin-mitophagy-lysosomal axis were quantified in the mid-intestine. While Parkin remained unchanged in ASD-like larvae (Figure 3B), both PINK1 and LC3B expression were significantly reduced (Figure 3A,E). In contrast, Rab 5 and LAMP1 expression were significantly increased (Figure 3C,D).

### 3.3. High-Resolution Mitochondrial Morphometrics Suggest a Fission Bias

**Figure 4:**
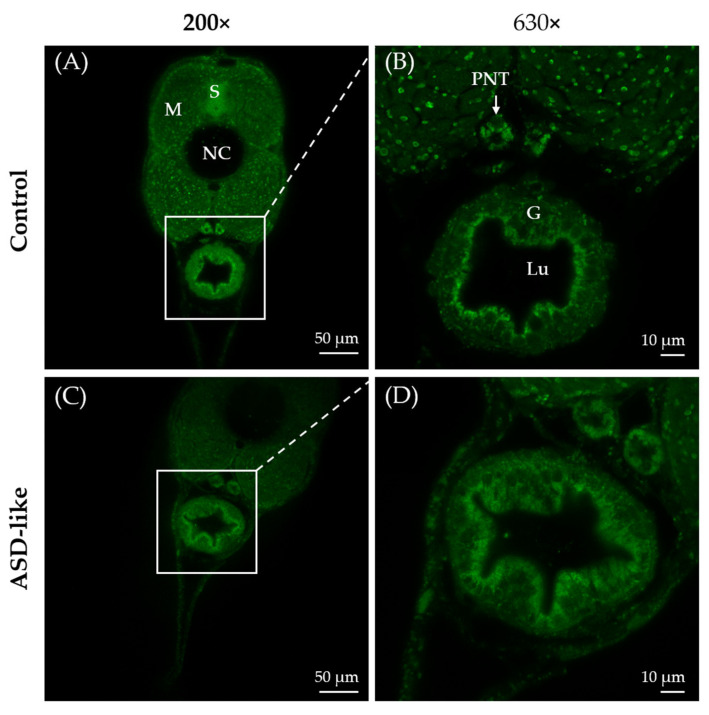
TOMM20 expression in an ASD-like zebrafish larval gut. Representative micrographs of mid-intestinal cross-sections of (**A**,**B**) control and (**C**,**D**) ASD-like zebrafish larvae. Captured at 200× ((**A**,**C**), scale bar = 50 µm) and 630× ((**B**,**D**), scale bar = 10 µm) magnification. Abbreviations: G, gut; Lu, lumen; M, muscle, NC, notochord; PNT, pronephros tubule; S, spinal cord.

In the gut, qualitative assessment suggested an increase in TOMM20 signal (200× and 630× magnification), and more diffuse distribution throughout the cytosol (Figure 4) in ASD-like larvae, while in control larvae, the TOMM20 signal was localized at the apical border.

**Figure 5:**
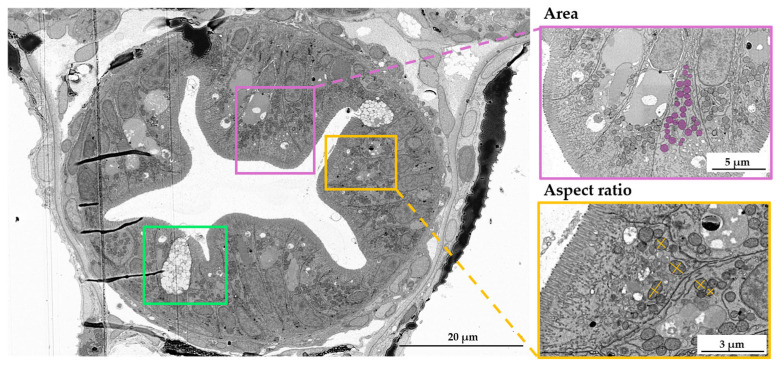
Representative electron micrographs indicating morphometric parameters assessed in zebrafish larval mid-intestinal regions. The purple block illustrates mitochondrial area, while the yellow block represents the mitochondrial aspect ratio. The green block highlights a goblet cell, primarily situated in the mid-intestinal region of the larval gut. Micrographs were captured at 1500× (scale bar = 20 µm), 6000× (scale bar = 5 µm) and 12,000× (scale bar = 3 µm).

Given the qualitative nature of the confocal observations, transmission electron microscopy (9000× magnification) was utilized to enable high-resolution assessment of mitochondrial ultrastructure, with a focus on morphometric parameters as illustrated in Figure 5. In short, the number of mitochondria (average visible mitochondria/10 cells), mitochondrial cross-sectional areas, and mitochondrial aspect ratios—calculated as the average ratio of the minor axis and the major axis per cell—were quantified.

**Figure 6:**
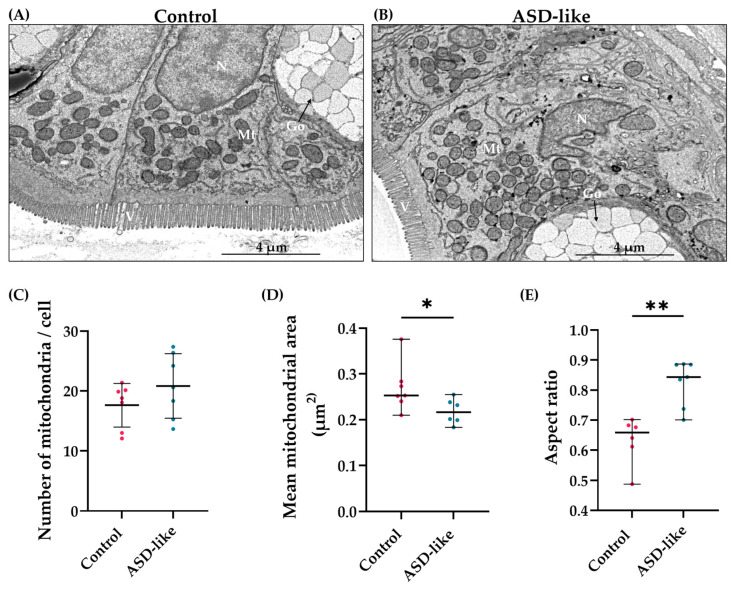
Morphometric analysis of mitochondria in an ASD-like zebrafish larval mid-intestine. Representative electron micrograph of (**A**) control and (**B**) ASD-like larvae. Images were captured at 9000× magnification (scale bar = 4 µm). Quantitative data are presented: (**C**) mitochondrial number per cell, (**D**) mitochondrial area, and (**E**) mitochondrial aspect ratio. In (**C**)data are presented as mean ± SD data, *n* = 6–7 with eachdata point representing the average number of mitochondria in 10 cells. In (**E**), data are presented as median with 95% CI, *n* = 6–8 per group where each *n* is the average of 60 individual mitochondria. Statistical analysis: Mann–Whitney U-test. * *p* < 0.05, ** *p* < 0.01. Abbreviations: Go, goblet cells; N, nucleus; Mt, mitochondria; V, villi.

At high resolution, potentially due to limited sample number, increases in the number of mitochondria per cell in the ASD-like group did not reach statistical significance (as in lower magnification, but higher sample size assessment of TOMM20). However, mitochondria in the gut of the ASD-like phenotype were significantly smaller and less elongated (Figure 6).

## 4. Discussion

Multimodal elucidation of the gut mitochondrial profile yielded several insights on mitochondrial processes that may underpin ASD-associated symptomology. Firstly, our data demonstrated ASD-associated redox imbalance and reduced mitochondrial respiration at a whole organism level. Secondly, in the gut specifically, high-resolution mitochondrial morphometrics indicated ASD-associated changes in mitochondrial morphology, organization and distribution, which may be a primary feature of ASD-like gut. Furthermore, in the gut context, alterations in the markers associated with the PINK1/Parkin mitophagy-lysosomal axis seem to suggest impaired mitochondrial recycling should be considered in the ASD context.

In the clinical setting, in terms of redox imbalance, several studies indicate decreased SOD levels in serum [40,41,42] and brain biopsies [25] from children with ASD compared to neurotypical controls. The decrease in overall SOD activity reported in the current study aligns with this. Similarly, the observed decrease in whole body non-enzymatic antioxidant capacity (TEAC) in ASD again aligns with clinical reports of dysregulation in the endogenous (glutathione) antioxidant system [43,44]. A strength of the current data is that the illustrated antioxidant deficits were linked to increased ROS.

**Figure 7:**
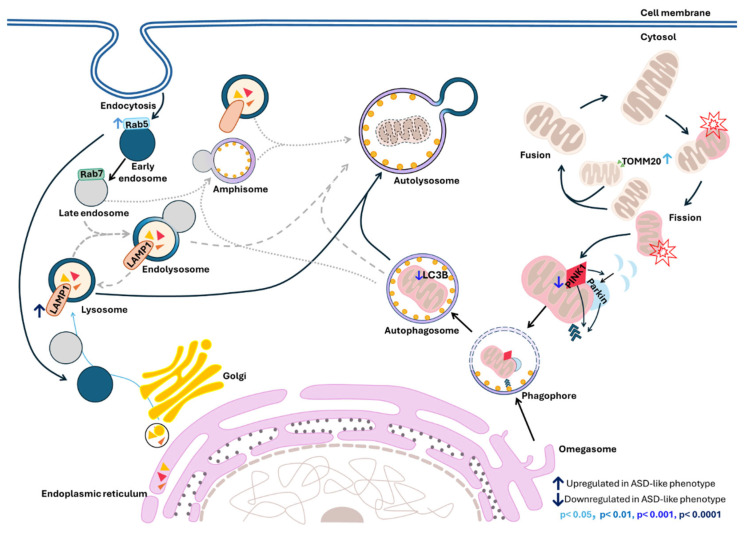
Overview of dysregulated mitochondrial recycling via the PINK1/Parkin-mitophagy-lysosomal axis in the ASD-like GIT epithelia. Mitochondrial dynamics comprise the coordinated processes of fusion, fission, and mitophagy that maintain mitochondrial quality and network integrity. Fusion enables the exchange of mitochondrial contents, whereas fission segregates damaged or dysfunctional components (red star) for removal. Following mitochondrial damage and depolarisation, PINK1 accumulates on the outer mitochondrial membrane, recruits Parkin, and promotes ubiquitination of mitochondrial proteins, targeting damaged mitochondria for autophagic degradation. The phagophore, an open cup-shaped isolation membrane, elongates and engulfs damaged mitochondria before sealing to form a double-membraned autophagosome marked by LC3-II (lapidated form of LC3B). In parallel, early endosomes (Rab5-positive), formed following plasma membrane fission during endocytosis, mature into late endosomes that deliver endocytic cargo to lysosomes via either direct fusion or transient “kiss-and-run” interactions. Late endosomes may also fuse with autophagosomes to form amphisomes prior to lysosomal fusion. Lysosomes (LAMP1-positive) subsequently fuse with the autophagosome to form autolysosomes, where mitochondrial cargo is degraded.

Turning attention to mitochondria as role player, our collective summary of proposed ASD-associated mitochondrial dysregulation the gut epithelium supported by current data, is presented in Figure 7. Mitochondrial TOMM20 expression was relatively increased in the ASD-like phenotype. Although no data on TOMM20 protein levels in the gut of humans with ASD is available, significantly increased TOMM20 protein levels have been reported in post-mortem brain temporal lobe samples from young children with ASD [25]. Interestingly, in the same study, TOMM20 levels seemed to decrease with age, with similar levels observed for control vs. ASD samples from adolescents, and a significantly decreased expression in ASD adults. The latter observation (albeit mRNA and not protein level) was also reported in other brain regions, such as the anterior cingulate gyrus (ACG) and motor cortex (MC) [45]. Further investigation in studies with longitudinal design—perhaps from larval to adult zebrafish—could confirm and elucidate the significance of an age factor in management of mitochondrial dysregulation in ASD.

High-resolution imaging elucidated the nature of mitochondrial morphometric dysregulation in the gut. Specifically, EM-based analysis illustrated smaller-sized and rounder-shaped gut mitochondria, suggestive of a morphological fission bias in the ASD-like phenotype. This novel result in the gut aligns with a previous report suggestive of a mitochondrial fission bias in the human ASD brain [25]. Furthermore, the fact that this fission bias persists across a lifespan [25], and does not seem compartment-specific, suggests it may be a primary pathology of ASD, rather than a maladaptive change, although this remains to be confirmed in large clinical cohorts. Given the increase in TOMM20 signal in the immunofluorescent analysis, we anticipated an increase in mitochondrial number in EM-based analysis. While current data did not achieve statistical significance between groups, an alternative explanation is that the immunofluorescent intensity reflected mitochondrial clustering rather than direct mitochondrial count, suggesting that these could be distinct biological signals. Future studies quantifying ultrastructural hallmarks of mitophagy (e.g., autolysosomes containing mitochondria) could confirm the proposed fission bias. For example, employing CLEM methodologies could confirm the relationship between TOMM20 signal and mitochondrial abundance as well as mitophagic structures. This would clarify if the proposed fission bias were coupled with mitophagic engulfment and identify potential targets for therapeutic modification.

In terms of mitochondrial function, the subtle decrease in whole body mitochondrial respiration observed in the current study aligns with data from another human report by Anitha and colleagues [46], which showed reduced Complex I (*NDUFA5*) and Complex V (*ATP5A1* and *ATP5G3*) gene expression in all assessed brain regions (AGC, MC and thalamus). However, in contrast, the single available report on mitochondrial function in the gut (of children with ASD) [23] demonstrated higher protein levels for ETC Complexes I, III, IV and V (in caecal but not rectal tissue), as well as relatively increased activity of ETC Complex I (in both rectal and caecal) tissue samples. These differences could relate to the fact that these gut regions most closely align with the posterior and not mid-intestinal regions of the zebrafish larval gut. The authors attributed this result to dysbiosis-related altered mitochondrial metabolism. However, given alignment of current results (generated in larvae where a microbiome is not yet a confounder) with the human brain data, the contrasting data reported by Rose and colleagues may suggest sample site specificity (gut region), highlighting the importance of considering metabolic activity when contextualizing data. Also, the different nature of dysregulation observed in different compartments, suggests that ETC dysregulation is likely an adaptive response, rather than a primary abnormality in ASD. A limitation in this context is that mitochondrial respiration in the current study was assessed at a whole organism level. While this clearly demonstrates systemic mitochondrial dysregulation, it does not directly reflect tissue (gut) level bioenergetics. Thus, the nature of the mitochondrial bioenergetic dysfunction and its contribution to ASD-associated gut symptomology remains to be elucidated.

Moreover, the current study has investigated an additional mechanism that could potentially contribute to persistent mitochondrial dysfunction in the gut—namely altered mitochondrial recycling. The increase in lysosomal abundance (NR positive stain) observed in the ASD-like mid-intestine was corroborated by a corresponding increase in LAMP1 expression. This aligns with a report of elevated *LAMP1* mRNA in whole blood samples from children with ASD, and bioinformatics data from the same study which supported an interpretation of upregulated blood and brain (cortex) using preclinical data [47]. Together, these data position LAMP1 as a potential target (diagnostic and/or monitoring) in the ASD context. To interpret this finding in a broader, functional context, the current report of increased Rab5 protein expression may suggest that an increased demand for mitochondrial recycling via endolysosomal activity could underpin the lysosomal accumulation suggested by elevated LAMP1 (and NR). In support of this interpretation, many endosomal pathway genes have been associated with ASD [48]; however, the current study is the first to investigate Rab5 in the ASD gut context. Nevertheless, this interpretation requires confirmation in purpose-designed experiments assessing lysosomal dynamics.

Turning attention to mitochondrial markers, observed deficits in the markers associated with the PINK1/Parkin-mitophagy-lysosomal axis in the current study may provide further insights. Although Parkin protein expression levels were not different in ASD vs. control, the ASD-like phenotype was associated with significantly decreased PINK1 expression, suggesting a relative reduction in damage sensing. In further support of potential impairment of canonical mitophagy, the observed decreased protein expression of LC3B could reflect reduced autophagophore formation and overall autophagy initiation. Such changes may be linked to hyperactivation of mTOR, as previously reported in ASD (both clinically and preclinically) [49,50,51]. Nevertheless, since LC3B levels are influenced by autophagic flux, this finding cannot distinguish between reduced formation and increased lysosomal turnover in the absence of flux assays. Collectively, while these data are consistent with, but not confirmatory of impaired canonical mitophagy and a potential compensatory rerouting toward alternative trafficking routes (endosomal pathways), definitive conclusions require flux assessments in this context.

In conclusion, the current study contributes to our understanding of the nature of mitochondrial dysregulation, both as potential primary pathology and adaptive dysregulation, in the ASD-like gut for the first time. Given the alignment with mitochondrial dysregulation reported clinically, current data confirms the accuracy of the VPA model to reflect mitochondrial abnormalities in an ASD-like context. Current data lay the basis for using this model to more comprehensively assess mechanisms underpinning mitochondrial and gut pathophysiology in the ASD context longitudinally. Downstream, this may allow for the development of intervention strategies either targeting primary mitochondrial pathology or supporting secondary maladaptive mitochondrial outcomes.

## Data Availability

The original contributions presented in this study are included in the article. Further enquiries can be directed to the corresponding author. The raw data supporting the conclusions of this article will be made available by the authors on request.

## Abbreviations

The following abbreviations are used in this manuscript:

Ama	Antimycin A
As	Ascorbate
ASD	Autism spectrum disorder
Azd	Sodium Azide
c	Cytochrome C
D	Adenosine diphosphate (ADP)
dpf	Days post fertilization
ETC	Electron transport chain
G	Glutamate
Gp	Glycerol phosphate
hpf	Hours post fertilization
LAMP1	Lysosome associated protein 1
LC3B	Microtubule-associated protein 1A/B-light chain 3 -phosphatidylethanolamine conjugate
Lu	Lumen
M	Malate
M	Muscle
NC	Notochord
Oct	Octanoylcarcinate
P	Pyruvate
PINK1	PTEN-induced kinase 1
PNT	Pronephros tubule
Rab5	Ras-associated protein 5
ROS	Reactive oxygen species
Rot	Rotenone
S	Succinate
SOD	Superoxide dismutase
SUIT	Substrate-uncoupler-inhibitor-titration
TEAC	Trolox equivalent antioxidant capacity
Tm	Tetramethyl phenylenediamine (TMPD)
U	Uncoupler (Carbonyl cyanide m-chlorophenylhydrazone)
VPA	Valproic acid

## Appendix A

**Table A1:** Summary of published VPA concentrations used to induce an ASD-like phenotype in zebrafish embryo and larvae, illustrating that the dose used in the current study, fits into the lower (subtoxic) end of the dose range.

Dose (µM)	Exposure (hpf)	Experimental Endpoint (dpf)	Reference
0.1, 0.3, 1, 3, 10, 30	6–48	2	[52]
0.5, 5, 15, 30, 50	4–120	6	[53]
1, 2.5, 5	5–24 and 5–48	21–28, 90	[54]
1, 5, 15, 25, 48, 75	4–120	7, 21, 42	[55]
5, 10, 20, 40, 80, 160, 320, 640 *, 1280 * and 2560 *	5–120	6	[56]
5, 50, 500, 1000 *, 1500 *	8–108	5, 10, 11, 12, 13	[57]
10, 20, 30 *, 40 *	8–108	5, 13, 90–120	[58]
25	10–24	2, 5–8, 30	[59]
25, 50	4–96	120	[60]
25, 50, 100	1–13 and 14–37	5	[61]
48	0–48	6, 30, 70,120	[11]
75	4–120	7/21	[10]
250	6–120	5	[62]

* Doses that induced major morphological defects and/or mortality. Abbreviations: hpf, hours post fertilization; dpf, days post fertilization.

**Table A2:** Concentrations of the substrates, uncouplers and inhibitors titrated to assess mitochondrial respiratory states.

Substrates, Uncouplers and Inhibitors *		Titration Vol. (µL)	Final Conc.	Respiratory State
Pyruvate	P	5	5 mM	Induce LEAK respiration
Malate	M	10	2 mM	
Adenosine diphosphate	ADP	10	1 mM	Induce complex I-linked OXPHOS
Cytochrome C	Cyt c	5	10 µM	To assess the integrity of the mitochondrial outer membrane
Carbonyl cyanide m-chlorophenylhydrazone (CCCP)	U	1	0.5 µM	Titrated in a stepwise manner to reach maximal respiratory levels
Glutamate	G	10	10 mM	Measures the contribution of the N-pathway to ETS capacity
Succinate	S	20	10 mM	Measure the contribution of S-pathway to ETS
Octanoylcarnitine	Oct	10	0.5 µM	Measure the contribution of the F-pathway to ETS
Rotenone	Rot	1	1 µM	Inhibit electron flow from the C-I pathway and measure the C-II-linked ETS capacity
Glycerophosphate	Gp	20	10 mM	Measure the contribution of Gp-pathway to ETS
Antimycin A	Ama	1	2.5 µM	To inhibit mitochondrial respiration and detect Rox due to oxidative side reactions.
tetramethyl-phenylenediamine (TMPD)	Tm	5	0.5 mM	Measure complex IV activity
Ascorbate	As	5	2 mM	To inhibit complex IV
Sodium azide	Azd	100	200 mM	

* All substrates and inhibitors used were purchased from Merck, Cape Town, South Africa. Abbreviations: conc., concentration; vol, volume.

## References

[1] Zeidan J., Fombonne E., Scorah J., Ibrahim A., Durkin M.S., Saxena S., Yusuf A., Shih A., Elsabbagh M. (2022). Global prevalence of autism: A systematic review update. Autism Res..

[2] Cheng N., Rho J.M., Masino S.A. (2017). Metabolic Dysfunction Underlying Autism Spectrum Disorder and Potential Treatment Approaches. Front. Mol. Neurosci..

[3] Madra M., Ringel R., Margolis K.G. (2020). Gastrointestinal Issues and Autism Spectrum Disorder. Child Adolesc. Psychiatr. Clin. N. Am..

[4] Bishop L., Charlton R.A., McLean K.J., McQuaid G.A., Lee N.R., Wallace G.L. (2023). Cardiovascular disease risk factors in autistic adults: The impact of sleep quality and antipsychotic medication use. Autism Res..

[5] Griborio-Guzman A.G., Ducas R.A. (2025). Autism and Its Correlation with Increased Cardiovascular Mortality and Diseases. Can. J. Cardiol..

[6] Li Y., Xie T., Li L., Lin J., Vos M., Chang Z., Snieder H., Hartman C.A. (2026). Cardiometabolic conditions in people with autism: A nationwide prospective cohort study from the Netherlands. Nat. Ment. Health.

[7] Zarate-Lopez D., Torres-Chávez A.L., Gálvez-Contreras A.Y., Gonzalez-Perez O. (2024). Three Decades of Valproate: A Current Model for Studying Autism Spectrum Disorder. Curr. Neuropharmacol..

[8] Hernández-Díaz S., Straub L., Bateman Brian T., Zhu Y., Mogun H., Wisner Katherine L., Gray Kathryn J., Lester B., McDougle Christopher J., DiCesare E. (2024). Risk of Autism after Prenatal Topiramate, Valproate, or Lamotrigine Exposure. N. Engl. J. Med..

[9] Ukezono M., Kasahara Y., Yoshida C., Murakami Y., Okada T., Takano Y. (2024). Impairments of social interaction in a valproic acid model in mice. Front. Behav. Neurosci..

[10] Dwivedi S., Medishetti R., Rani R., Sevilimedu A., Kulkarni P., Yogeeswari P. (2019). Larval zebrafish model for studying the effects of valproic acid on neurodevelopment: An approach towards modeling autism. J. Pharmacol. Toxicol. Methods.

[11] Zimmermann F., Gaspary K., Leite C., Cognato G., Bonan C. (2015). Embryological exposure to valproic acid induces social interaction deficits in zebrafish (*Danio rerio*): A developmental behavior analysis. Neurotoxicol. Teratol..

[12] Hirsch M.M., Deckmann I., Santos-Terra J., Staevie G.Z., Fontes-Dutra M., Carello-Collar G., Körbes-Rockenbach M., Brum Schwingel G., Bauer-Negrini G., Rabelo B. (2020). Effects of single-dose antipurinergic therapy on behavioral and molecular alterations in the valproic acid-induced animal model of autism. Neuropharmacology.

[13] Camussi D., Naef V., Brogi L., Della Vecchia S., Marchese M., Nicoletti F., Santorelli F.M., Licitra R. (2024). Delving into the Complexity of Valproate-Induced Autism Spectrum Disorder: The Use of Zebrafish Models. Cells.

[14] Prince P.D., Codagnone M.G., Opezzo J.A.W., Adán Areán J.S., Höcht C., Arnal N., Alvarez S., Zárate S., Reinés A. (2026). Alteration in hippocampal mitochondria ultrastructure and cholesterol accumulation linked to mitochondrial dysfunction in the valproic acid rat model of autism spectrum disorders. Psychopharmacology.

[15] Pretorius L., Coetzee J.A., Theron J., Latakgomo A., Smith C. (2026). Expanded characterization of the valproic acid zebrafish larval model of autism spectrum disorder: Insights into dysregulation in the gut. Anim. Model. Exp. Med..

[16] Varley A.N., Browning K.N. (2024). Gastrointestinal dysfunction in the valproic acid induced model of social deficit in rats. Auton. Neurosci..

[17] Restrepo B., Taylor S.L., Dominic Ponzini M., Angkustsiri K., Solomon M., Rogers S.J., Ashwood P., Say D.S., Caceres S., Alavynejad S. (2025). A longitudinal evaluation of gastrointestinal symptoms in children with autism spectrum disorder. Autism.

[18] Wang J., Ma B., Wang J., Zhang Z., Chen O. (2022). Global prevalence of autism spectrum disorder and its gastrointestinal symptoms: A systematic review and meta-analysis. Front. Psychiatry.

[19] Haque P.S., Kapur N., Barrett T.A., Theiss A.L. (2024). Mitochondrial function and gastrointestinal diseases. Nat. Rev. Gastroenterol. Hepatol..

[20] Fu S.-C., Qu J.-Y., Li L.-X., Yang X.-X., Li Y.-Q., Zuo X.-L. (2023). Excessive Mitochondrial Fission Suppresses Mucosal Repair by Impairing Butyrate Metabolism in Colonocytes. Inflamm. Bowel Dis..

[21] Diquigiovanni C., Rizzardi N., Cataldi-Stagetti E., Gozzellino L., Isidori F., Valenti F., Orsini A., Astolfi A., Giangregorio T., Pironi L. (2025). Glutamine Supplementation as a Novel Metabolic Therapeutic Strategy for LIG3-Dependent Chronic Intestinal Pseudo-Obstruction. Gastroenterology.

[22] Peña-Cearra A., Song D., Castelo J., Palacios A., Lavín J.L., Azkargorta M., Elortza F., Fuertes M., Pascual-Itoiz M.A., Barriales D. (2023). Mitochondrial dysfunction promotes microbial composition that negatively impacts on ulcerative colitis development and progression. NPJ Biofilms Microbiomes.

[23] Rose S., Bennuri S.C., Murray K.F., Buie T., Winter H., Frye R.E. (2017). Mitochondrial dysfunction in the gastrointestinal mucosa of children with autism: A blinded case-control study. PLoS ONE.

[24] Rossignol D.A., Frye R.E. (2012). Mitochondrial dysfunction in autism spectrum disorders: A systematic review and meta-analysis. Mol. Psychiatry.

[25] Tang G., Gutierrez Rios P., Kuo S.H., Akman H.O., Rosoklija G., Tanji K., Dwork A., Schon E.A., Dimauro S., Goldman J. (2013). Mitochondrial abnormalities in temporal lobe of autistic brain. Neurobiol. Dis..

[26] Chauhan A., Gu F., Essa M.M., Wegiel J., Kaur K., Brown W.T., Chauhan V. (2011). Brain region-specific deficit in mitochondrial electron transport chain complexes in children with autism. J. Neurochem..

[27] Weissman J.R., Kelley R.I., Bauman M.L., Cohen B.H., Murray K.F., Mitchell R.L., Kern R.L., Natowicz M.R. (2008). Mitochondrial Disease in Autism Spectrum Disorder Patients: A Cohort Analysis. PLoS ONE.

[28] Goldenthal M.J., Damle S., Sheth S., Shah N., Melvin J., Jethva R., Hardison H., Marks H., Legido A. (2015). Mitochondrial Enzyme Dysfunction in Autism Spectrum Disorders; A Novel Biomarker Revealed From Buccal Swab Analysis. Biomark. Med..

[29] Delhey L., Kilinc E.N., Yin L., Slattery J., Tippett M., Wynne R., Rose S., Kahler S., Damle S., Legido A. (2017). Bioenergetic variation is related to autism symptomatology. Metab. Brain Dis..

[30] Chen S., Li Z., He Y., Zhang F., Li H., Liao Y., Wei Z., Wan G., Xiang X., Hu M. (2015). Elevated mitochondrial DNA copy number in peripheral blood cells is associated with childhood autism. BMC Psychiatry.

[31] Napoli E., Wong S., Hertz-Picciotto I., Giulivi C. (2014). Deficits in bioenergetics and impaired immune response in granulocytes from children with autism. Pediatrics.

[32] Giulivi C., Zhang Y.F., Omanska-Klusek A., Ross-Inta C., Wong S., Hertz-Picciotto I., Tassone F., Pessah I.N. (2010). Mitochondrial Dysfunction in Autism. JAMA.

[33] Frye R.E., Rincon N., McCarty P.J., Brister D., Scheck A.C., Rossignol D.A. (2024). Biomarkers of mitochondrial dysfunction in autism spectrum disorder: A systematic review and meta-analysis. Neurobiol. Dis..

[34] Pretorius L., Moodley T., Smith C. (2026). Sex-specific neurological dysregulation may underpin distinctly different male and female behavioural phenotypes in a zebrafish model of autism spectrum disorder. Behav. Brain Funct..

[35] Pretorius L., Ross K.S., Smith C. (2025). Multi-targeted action of rooibos may protect against ischaemic stroke-induced neurological deficit and endothelial dysfunction. J. Ethnopharmacol..

[36] Pretorius L., Smith C. (2022). Aspalathus linearis (Rooibos) and Agmatine May Act Synergistically to Beneficially Modulate Intestinal Tight Junction Integrity and Inflammatory Profile. Pharmaceuticals.

[37] Deerinck T.J., Bushong E., Thor A., Ellisman M. (2010). NCMIR methods for 3D EM: A new protocol for preparation of biological specimens for serial block face scanning electron microscopy. Nat. Center Microsc. Imag. Res..

[38] Morales Fénero C., Amaral M.A., Xavier I.K., Padovani B.N., Paredes L.C., Takiishi T., Lopes-Ferreira M., Lima C., Colombo A., Saraiva Câmara N.O. (2021). Short chain fatty acids (SCFAs) improves TNBS-induced colitis in zebrafish. Curr. Res. Immunol..

[39] Li I.C., Chan C.T., Lu Y.F., Wu Y.T., Chen Y.C., Li G.B., Lin C.Y., Hwang S.P. (2011). Zebrafish krüppel-like factor 4a represses intestinal cell proliferation and promotes differentiation of intestinal cell lineages. PLoS ONE.

[40] Shareef A.A., Kheder A.H., Albarzinji N., Karim K.J., Smail S.W., Mahmood A.A., Amin K. (2025). Oxidative markers and SOD variant: Predictors of autism severity and susceptibility. Future Sci. OA.

[41] Afrazeh M., Saedisar S., Khakzad M.R., Hojati M. (2015). Measurement of Serum Superoxide Dismutase and Its Relevance to Disease Intensity Autistic Children. Maedica.

[42] Wang L., Jia J., Zhang J., Li K. (2016). Serum levels of SOD and risk of autism spectrum disorder: A case-control study. Int. J. Dev. Neurosci..

[43] Frustaci A., Neri M., Cesario A., Adams J.B., Domenici E., Dalla Bernardina B., Bonassi S. (2012). Oxidative stress-related biomarkers in autism: Systematic review and meta-analyses. Free Radic. Biol. Med..

[44] Bjørklund G., Tinkov A.A., Hosnedlová B., Kizek R., Ajsuvakova O.P., Chirumbolo S., Skalnaya M.G., Peana M., Dadar M., El-Ansary A. (2020). The role of glutathione redox imbalance in autism spectrum disorder: A review. Free Radic. Biol. Med..

[45] Anitha A., Nakamura K., Thanseem I., Yamada K., Iwayama Y., Toyota T., Matsuzaki H., Miyachi T., Yamada S., Tsujii M. (2012). Brain region-specific altered expression and association of mitochondria-related genes in autism. Mol. Autism.

[46] Anitha A., Nakamura K., Thanseem I., Matsuzaki H., Miyachi T., Tsujii M., Iwata Y., Suzuki K., Sugiyama T., Mori N. (2013). Downregulation of the expression of mitochondrial electron transport complex genes in autism brains. Brain Pathol..

[47] Deng S., Feng X., Yang M., Yu W., Wu Z., Zhu X., Song Z., Cheng S. (2023). LAMP1 as a novel molecular biomarker to predict the prognosis of the children with autism spectrum disorder using bioinformatics approaches. Sci. Rep..

[48] Patak J., Zhang-James Y., Faraone S.V. (2016). Endosomal system genetics and autism spectrum disorders: A literature review. Neurosci. Biobehav. Rev..

[49] Crespi B.J. (2019). Comparative psychopharmacology of autism and psychotic-affective disorders suggests new targets for treatment. Evol. Med. Public Health.

[50] Nicolini C., Ahn Y., Michalski B., Rho J.M., Fahnestock M. (2015). Decreased mTOR signaling pathway in human idiopathic autism and in rats exposed to valproic acid. Acta Neuropathol. Commun..

[51] Qin L., Dai X., Yin Y. (2016). Valproic acid exposure sequentially activates Wnt and mTOR pathways in rats. Mol. Cell. Neurosci..

[52] Muhsen M., Youngs J., Riu A., Gustafsson J.-Å., Kondamadugu V.S., Garyfalidis E., Bondesson M. (2021). Folic acid supplementation rescues valproic acid-induced developmental neurotoxicity and behavioral alterations in zebrafish embryos. Epilepsia.

[53] Bailey J.M., Oliveri A.N., Karbhari N., Brooks R.A.J., De La Rocha A.J., Janardhan S., Levin E.D. (2016). Persistent behavioral effects following early life exposure to retinoic acid or valproic acid in zebrafish. NeuroToxicology.

[54] Messina A., Sovrano V.A., Baratti G., Musa A., Gobbo A., Adiletta A., Sgadò P. (2024). Valproic acid exposure affects social visual lateralization and asymmetric gene expression in zebrafish larvae. Sci. Rep..

[55] Karimi Z., Zarifkar A., Dianatpour M., Mirzaei E., Dara M., Aligholi H. (2023). Finding a Proper Valproic Acid-Based Autism Spectrum Disorder Model in Zebrafish: Early and Long-term Neurobehavioral Studies. Iran. J. Psychiatry Behav. Sci..

[56] Saleh Hodin N.A., Chong S.G., Bakar N.A., Fahmi M.S.A.M., Ramlan N.F., Hamid N.N.A.Z.Z., Fadzar M.S.I.M., Zulkifli A.R., Norazhar A.I., Mastuki S.N. (2023). Toxicity and teratogenicity effects of valproic acid on zebrafish (*Danio rerio*) embryos in relation to autism spectrum disorder. Birth Defects Res..

[57] Chen J., Lei L., Tian L., Hou F., Roper C., Ge X., Zhao Y., Chen Y., Dong Q., Tanguay R.L. (2018). Developmental and behavioral alterations in zebrafish embryonically exposed to valproic acid (VPA): An aquatic model for autism. Neurotoxicol. Teratol..

[58] Joseph T.P., Zhou F., Sai L.Y., Chen H., Lin S.L., Schachner M. (2022). Duloxetine ameliorates valproic acid-induced hyperactivity, anxiety-like behavior, and social interaction deficits in zebrafish. Autism Res..

[59] DeOliveira-Mello L., Baronio D., Panula P. (2023). Zebrafish embryonically exposed to valproic acid present impaired retinal development and sleep behavior. Autism Res..

[60] Wang J., Zou L., Jiang P., Yao M., Xu Q., Hong Q., Zhu J., Chi X. (2024). Vitamin A ameliorates valproic acid-induced autism-like symptoms in developing zebrafish larvae by attenuating oxidative stress and apoptosis. NeuroToxicology.

[61] Gebuijs I.G.E., Metz J.R., Zethof J., Carels C.E.L., Wagener F.A.D.T.G., Von den Hoff J.W. (2020). The anti-epileptic drug valproic acid causes malformations in the developing craniofacial skeleton of zebrafish larvae. Mech. Dev..

[62] Dixon S.C., Calder B.J., Lilya S.M., Davies B.M., Martin A., Peterson M., Hansen J.M., Suli A. (2023). Valproic acid affects neurogenesis during early optic tectum development in zebrafish. Biol. Open.

